# Infectious complications in CLL/SLL patients receiving Bruton's Tyrosine Kinase inhibitors – systematic review and meta-analysis of randomized controlled trials

**DOI:** 10.1007/s00277-025-06502-y

**Published:** 2025-07-25

**Authors:** Shira Buchrits, Gal Trieman, Odil Giladi, Liron Hofstetter, Ronit Gurion, Galit Granot, Adi Shacham-Abulafia, Pia Raanani, Anat Gafter-Gvili

**Affiliations:** 1 Institute of Hematology, Davidoff Cancer Center, Beilinson Hospital, Rabin Medical Center, 39 Jabotinsky Road, 49100 Petah Tikva, Israel; 2 Internal Medicine Department A, Beilinson Hospital, Rabin Medical Center, Petah Tikva, Israel; 3 Felsenstein Medical Research Center, Rabin Medical Center, Petah Tikva, Israel; 4 Faculty of Medical and Health Sciences, Tel Aviv University, Tel-Aviv, Israel

**Keywords:** Chronic lymphocytic leukaemia (CLL), BTK inhibitor toxicity, Bruton tyrosine kinase inhibitors

## Abstract

The development of Bruton Tyrosine Kinase inhibitors (BTKis) has revolutionized the management of chronic lymphocytic leukemia/small lymphocytic lymphoma (CLL/SLL). However, increased infection rates have been reported in patients receiving BTKis in multiple clinical trials. This study aimed to evaluate the risk of infections associated with BTKis compared to other therapeutic regimens in CLL/SLL patients. A systematic review and meta-analysis of randomized controlled trials (RCTs) was conducted. We included trials comparing BTKi-containing regimens (e.g., BTKi alone or combined with anti-CD20 or venetoclax) to other therapeutic regimens, as well as studies comparing different BTKis or BTKi combinations. Primary outcomes were the risk of any infection and grade 3–4 infections. Secondary outcomes included pneumonia, sepsis, septic shock, COVID-19, fungal infections, fatal infections, bacteremia, and febrile neutropenia. Pooled risk ratios (RR) with 95% confidence intervals (CIs) were estimated using fixed or random effects models based on heterogeneity. Eighteen trials encompassing 8,324 patients were included. BTKi-containing regimens, either as monotherapy or combined with anti-CD20 or venetoclax, were not associated with a significantly increased risk of any infection compared to other regimens [BTKi + anti-CD20 or venetoclax: RR 0.93 (95% CI: 0.79–1.09, I^2^ = 46%), 3 trials; BTKi monotherapy: RR 1.12 (95% CI: 0.94–1.34, I^2^ = 73%), 3 trials]. Similarly, BTKi monotherapy was not associated with an increased risk of grade 3–4 infections [RR 1.05 (95% CI: 0.76–1.44, I^2^ = 61%), 5 trials]. The risk of sepsis was not significantly increased with BTKi regimens [BTKi + anti-CD20: RR 0.48 (95% CI: 0.12–1.84, I^2^ = 69%), 4 trials; BTKi monotherapy: RR 0.50 (95% CI: 0.25–1.01, I^2^ = 0%), 5 trials]. However, pneumonia risk was increased in the BTKi + anti-CD20 vs other regimens, [RR 2.18; 95% CI 1.29–3.70; I^2^ = 3%, 4 trials]. The risk of febrile neutropenia was reduced in trials comparing BTKi-containing regimens to other therapies. BTKi-containing regimens were not associated with an increased risk of overall infections or grade 3–4 infections compared to other regimens. However, an elevated risk of pneumonia was observed with BTKi combinations, highlighting the need for careful consideration when selecting treatment regimens for CLL/SLL patients.

**Supplementary Information:**

The online version contains supplementary material available at 10.1007/s00277-025-06502-y.

## Introduction

Chronic lymphocytic leukemia (CLL) and its nodal variant, small lymphocytic lymphoma (SLL), are the most common types of leukemia in Western countries [1, 2]. Bruton’s tyrosine kinase (BTK) plays an important role in the proliferation and survival of malignant B lymphocyte in CLL [3]. The development of BTK inhibitors (BTKis) has transformed the management of CLL, both as a first-line treatment and in relapsed or refractory settings [4–7].

Ibrutinib, the first irreversible BTKi, was approved by the U.S. Food and Drug Administration in 2013, marking a major advance in CLL therapy [5]. Subsequently, second-generation covalent BTKis, such as acalabrutinib and zanubrutinib, were developed to enhance efficacy while potentially offering a more favorable safety profile compared with ibrutinib, as reflected in the ELEVATE-RR and ALPINE trials [6, 7]. Additional irreversible BTKis are under investigation, alongside newer reversible BTKis, such as pirtobrutinib and vecabrutinib, which bind non-covalently to BTK. Notably, in the BRUIN-CLL-321 phase 3 trial, pirtobrutinib showed superior PFS versus standard therapy in BTKi-pretreated patients [8].

Bruton’s tyrosine kinase plays an important role in immunity, participating in numerous proliferation and survival pathways in cells such as B cells, T cells, and macrophages [3]. Previous observational studies and pooled safety analyses have reported an increased risk of infections among patients treated with BTKis. For example, in a safety analysis of four CLL/SLL trials involving 756 patients treated with ibrutinib, 70% developed any infection, with pneumonia being one of the most common grade 3/4 adverse events [9]. Moreover, a retrospective analysis of 308 patients with various B-cell malignancies treated with ibrutinib across four sequential, 45 patients discontinued treatment for reasons unrelated to progression or relapse, of whom 62% discontinued due to infections [10].

A systematic review of prospective studies reported that infections occurred in 56% of patients receiving single-agent ibrutinib and 52% of those on combination therapy. Approximately 20% of patients developed pneumonia, many of which were opportunistic [11].

Since CLL is associated with immune dysfunction and a high baseline risk of infections, comparing BTKi-based regimens to other treatments is necessary in an attempt to isolate the therapy’s specific impact on infectious outcomes. Given that new randomized controlled trials (RCTs) have since been published, we conducted this systematic review and meta-analysis to evaluate the risk of infections associated with BTKis compared to other therapeutic regimens in patients with CLL/SLL.

## Methods

### Data source

We conducted a systematic search to identify both published and unpublished randomized controlled trials (RCTs) evaluating BTKi-containing regimens for CLL/SLL. The following databases and sources were searched: PubMed (January 1966 to June 2025), the Cochrane Central Register of Controlled Trials (CENTRAL) (The Cochrane Library, Issue 7 of 12, July 2024), and the conference proceedings of major hematology meetings (2020–2025), including the Annual Meetings of the American Society of Hematology (ASH) and the European Hematology Association (EHA).

The PubMed and Cochrane searches included the terms: *(Chronic lymphocytic leukemia OR CLL OR Chronic lymphatic leukemia OR small lymphocytic lymphoma OR SLL) AND (Bruton’s tyrosine kinase inhibitors OR BTK inhibitors OR ibrutinib OR acalabrutinib OR zanubrutinib OR pirtobrutinib)*. Conference proceedings were searched using the term *“Bruton’s tyrosine kinase.”* Additionally, the references of all included trials and reviews were screened to identify further eligible studies.

### Study selection

We included all RCTs that compared BTKi-containing regimens, either as monotherapy or in combination with other agents, for the treatment of patients with CLL/SLL. Studies were eligible regardless of patients’ treatment lines (first-line therapy or relapsed/refractory disease), publication status, release date, or language. References from all included trials and identified reviews were also scanned for additional relevant studies.

### Data extraction and quality assessment

Two independent reviewers (SB, GT) extracted data from the included trials and assessed the quality of the methodologies. Discrepancies were resolved by a third evaluator (AGG), and final decisions were made by consensus. Potential sources of bias were evaluated, including allocation concealment, random sequence generation, blinding, incomplete outcome reporting, and selective outcome reporting. Each domain was rated as low risk, unclear risk (due to insufficient information), or high risk of bias, following the Cochrane Handbook for Systematic Reviews of Interventions (version 5.1.0) [12].

### Definition of outcomes

The primary outcome was the risk of any infection and the risk of grade 3–4 infection. Secondary outcomes included the following adverse events: pneumonia, sepsis, septic shock, COVID-19 infection, fungal infection, fatal infection, bacteremia and neutropenic fever, as defined in each trial.

### Data synthesis and analysis

We conducted seven comparisons:

1. BTKi + anti-CD20 *vs* another comparator.
2. BTKi + venetoclax *vs* another comparator.
3. BTKi + anti-CD20 + venetoclax *vs* another comparator.
4. BTKi monotherapy *vs* another comparator.
5. Ibrutinib *vs* other BTKis.
6. BTKi *vs* BTKi + anti-CD20.
7. BTKi + chemotherapy *vs* chemotherapy.

Dichotomous data were analyzed using risk ratios (RR) with 95% confidence intervals (CIs) calculated for each trial (Review Manager [RevMan], version 5.4). Heterogeneity was assessed using the χ^2^ test and the I^2^ statistic, with significant heterogeneity defined as I^2^ > 50%. For meta-analyses, we used the Mantel–Haenszel method for the fixed-effect model, while a random-effects model (REM) was applied when significant heterogeneity was detected. A risk ratio (RR) below 1 favors BTKi (lower infection risk), a value above 1 indicates higher risk compared to the control arm.

We aimed to conduct subgroup analyses of the primary outcomes to explore potential sources of heterogeneity based on BTKi type and therapeutic line (first-line versus relapsed/refractory disease). Results of the meta-analyses were graphically presented as forest plots.

## Results

### Description of included studies

**Fig. 1:**
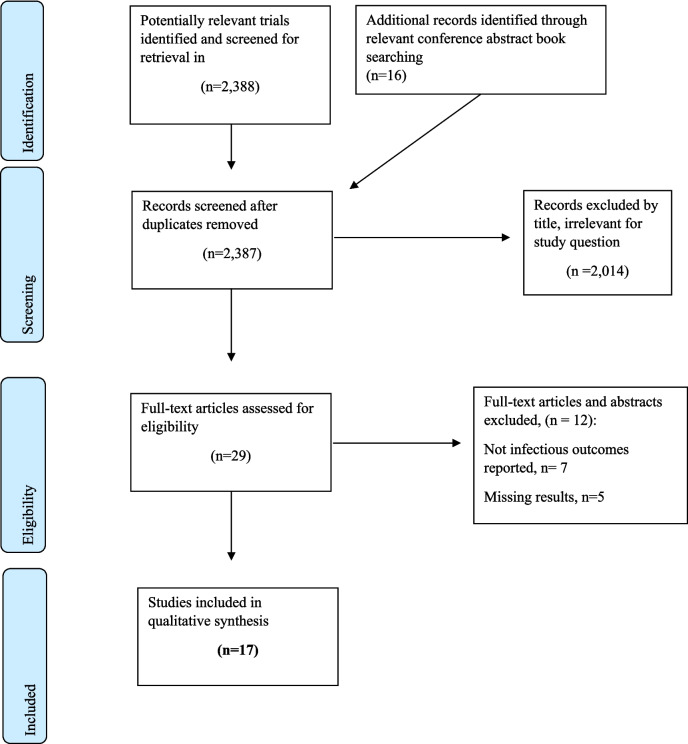
Trial flow according to preferred reporting items for systematic reviews and meta-analyses (PRISMA), showing flow of trials included in the meta-analysis

The search identified 2,659 potentially relevant publications, of which 30 were shortlisted for further investigation. Of these, 12 trials were excluded due to the absence of relevant data. Ultimately, 18 studies were included in the systematic review, comprising a total of 8,324 patients [4–7, 13–26]. A PRISMA flowchart (Fig. 1) illustrates the selection process, including reasons for exclusion.

**Fig. 2:**
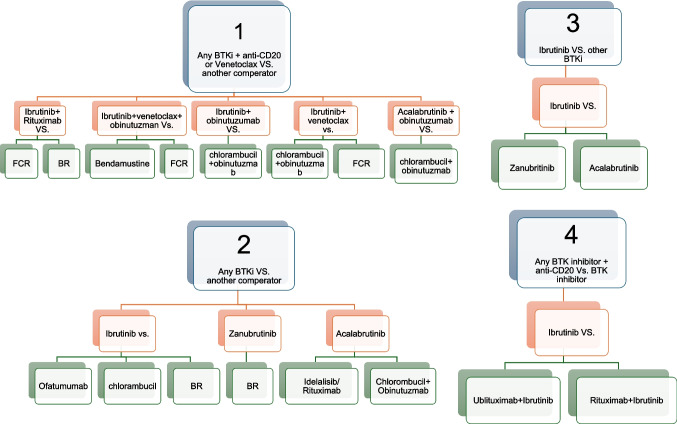
Chart of the included trials divided into four groups

The included trials evaluated various treatment combinations and were categorized into seven comparison groups, as shown in Fig. 2 and Table 1:

1. BTKi + anti-CD20 *vs* chemotherapy or chemoimmunotherapy (5 trials): Comparators included fludarabine + cyclophosphamide + rituximab (FCR, 3 trials: Alliance A041202 [20], E1912 [24], GENUINE [22]), bendamustine + rituximab (BR, 1 trial), and chlorambucil + obinutuzumab (2 trials: ELEVATE-TN [14], iLLUMINATE [23]).
2. BTKi + venetoclax *vs* chemotherapy or chemoimmunotherapy (4 trials): Comparators included FCR (2 trials: GAIA [17], FLAIR [19]), BR (1 trial: AMPLIFY [26]), and chlorambucil + obinutuzumab (1 trial: GLOW [18]).
3. BTKi + anti-CD20 + venetoclax *vs* chemotherapy or chemoimmunotherapy (2 trials): Comparators included FCR or BR (1 trial: GAIA [17]) and FCR or bendamustine + cyclophosphamide + rituximab (BCR, 1 trial: FLAIR [19]).
4. BTKi monotherapy vs. another comparator (6 trials): Comparators included ofatumumab (1 trial: RESONATE [5]), idelalisib + rituximab (1 trial: ASCEND [16]), chlorambucil (1 trial: RESONATE-2 [4]), chlorambucil + obinutuzumab (1 trial: ELEVATE-TN [14]), and BR (2 trials: Alliance A041202 [24], GENUINE [22]).
5. Ibrutinib vs. other BTKis (2 trials): Comparisons were made with zanubrutinib (1 trial: ALPINE [7]) and acalabrutinib (1 trial: ELEVATE-RR [6]).
6. BTKi vs. BTKi + anti-CD20 (4 trials): Comparators included ublituximab + ibrutinib (1 trial: GENUINE [22]), rituximab + ibrutinib (2 trials: Burger et al. [21], Alliance A041202 [20]), and obinutuzumab + acalabrutinib (1 trial: ELEVATE-TN [14]).
7. BTKi + chemotherapy vs chemotherapy alone (1 trial): One trial compared ibrutinib + BR vs BR (HELIOS [25]).

**Table 1:** Characteristics of included trials

	Study	Treatment arms	Mode of administration	Number of patients randomized	Inclusion criteria	Binet C
BTKi plus anti- CD20 vs. any other treatment	Hillmen (FLAIR), 2023 [16]	Ibrutinib^1^ + rituximab^2^	P.O + I.V	327	First line treatment	178/386, 46%
		FCR^3^	I.V	267		170/385, 44%
	Moreno (iLLUMINATE), 2019 [23]	Ibrutinib^1^ + obinutuzumab^4^	P.O + I.V	113	First line treatment	60/113, 53%
		Chlorambucil^5^ + obinutuzumab^4^	P.O + I.V	116		59/116, 51%
	Shanafelt (E1912), 2018 [24]	Ibrutinib^1^ + rituximab^2^	P.O + I.V	332	First line treatment	156/354, 44.1%
		FCR^3^	P.O + I.V	151		72/175, 41.1%
	Woyach (Alliance A041202), 2018 [20]	Ibrutinib^1^ + rituximab^2^	P.O + I.V	181	First line treatment	98/182, 54%
		Bendamustine^6^ + rituximab^2^	I.V	176		83/183, 45%
	Sharman (ELEVATE-TN), 2021 [14]	Acalabrutinib^7^ + obinutuzumab^4^	P.O + I.V	142	First line treatment	38/179, 21.2%
		Obinutuzumab^4^ + chlorambucil^5^	I.V	137		38/177, 21.5%
BTKi plus venetoclax vs. any other treatment	Niemann (GLOW), 2023 [18]	Ibrutinib^1^ + venetoclax^8^	P.O	106	First line treatment	43/96, 44.8%
		Obinutuzumab^4^ + chlorambucil^5^	P.O + I.V	105		40/101, 39.6
	Mohty (AMPLIFY), 2025 [26]	Acalabrutinib^7^ + venetoclax^8^	P.O	284	First line treatment	137/291, 47.1%
		FCR^3^/BR^10^	I.V	259		127/290, 43.8%
	Munir (FLAIR), 2023 [9]	Ibrutinib^1^ + venetoclax^8^	P.O	252	First line treatment	109/260, 41.9%
		FCR^3^	P.O + I.V	239		111/260, 42.2%
BTKi plus venetoclax plus anti CD-20 vs. any other treatment	Fürstenau (GAIA), 2024 [17]	Ibrutinib^1^ + venetoclax^8^ + obinutuzumab^4^	P.O + I.V	231	First line treatment	82/229, 35.8%
		BCR^9^/FCR^3^	I.V	216		84/231, 36.4%
	Mohty (AMPLIFY), 2025 [26]	Acalabrutinib^7^ + venetoclax^8^ + obinutuzumab^4^	P.O + I.V	284	First line treatment	116/286, 40.6%
		FCR^3^/BR^10^	I.V	259		127/290, 43.8%
BTKi monotherapyvs. any other treatment	Byrd (RESONATE), 2014 [5]	Ibrutinib^1^	P.O	195	R/R	102/195, 52%
		Oftatumumab^11^	I.V	196		104/196, 53%
	Burger (RESONATE 2), 2015 [4]	Ibrutinib^1^	P.O	131	First line treatment	60/136, 44%
		Chlorambucil^5^	I.V	114		62/133, 47%
	Tam (SEQUOIA), 2022 [13]	Zanubrutinib17 p deletion^12^	P.O	93	First line treatment	39/111, 35%
		Zanubrutinib^13^	P.O	206		70/241, 29%
		Bendamustine^6^ + rituximab^2^	I.V	188		70/238, 29%
	Ghia (ASCEND), 2020 [15]	Acalabrutinib^7^	P.O	124	R/R	65/155, 42%
		Investigator's choice (IR)^14^	P.O + I.V	38		64/155, 41%
		Investigator's choice (BR)^15^	I.V			
	Sharman (ELEVATE-TN), 2021 [14]	Acalabrutinib^7^	P.O	143	First line treatment	37/179, 20.7%
		Obinutuzumab^4^ + chlorambucil^5^	I.V	137		38/177, 21.5%
	Woyach (ALLIANCE), 2018 [20]	Bendamustine^6^ + rituximab^2^	I.V	176	First line treatment	83/183, 45%
		Ibrutinib^1^	P.O	180		79/182, 43%
Ibrutinib Vs. other BTKi	Brown (ALPINE), 2023 [7]	Ibrutinib^1^	P.O	240	R/R	145/327 44.3%
		Zanubrutinib^13^	P.O	260		135/325 41.5%
	Byrd (ELEVATERR), 2021 [6]	Ibrutinib^1^	P.O	109	R/R	104/268 38.8%
		Acalabrutinib^7^	P.O	124		114/265, 43%
BTKi Vs. BTKi with anti CD-20	Sharman (GENUINE), 2021 [22]	Ibrutinib^1^	P.O	58	R/R	26/59, 44%
		Ibrutinib^1^ + ublituximab^16^	P.O + I.V	59		31/61, 51%
	Burger, 2019 [21]	Ibrutinib^1^	P.O	104	R/R	38/104, 37%
		Ibrutinib^1^ + rituximab^2^	P.O + I.V	104		42/104, 40%
	Woyach (ALLIANCE), 2018 [20]	Ibrutinib^1^	P.O	180	First line treatment	99/182, 54%
		Ibrutinib^1^ + rituximab^2^	P.O + I.V	181		98/182, 54%
Chemotherapy + BTKI Vs. Chemotherapy	Chanan- Khan (HELIOS), 2016 [25]	Ibrutinib^1^+BR^10^	P.O + I.V	203	R/R	98/258, 38%
		Placebo^17^ + BR^10^		100		116/258, 45%

Abbreviations: *BCR* bendamustine + cyclophosphamide + rituximab, *BR* bendamustine + rituximab, *CLL* Chronic lymphocytic leukemia, *ECOG* Eastern Cooperative Oncology Group, *FCR* Fludarabine + cyclophosphamide + rituximab, *IR* idelalisib + rituximab, *I.V* intravenous, *P.O* per oss, *R/R* relapsed or refractory, *SLL* Small Lymphocytic Lymphoma

^1^Ibrutinib—420 mg once a day

^2^Rituximab—375 mg/m^2^ on day 1 of cycle 1 and 500 mg/m^2^ on day 1 in cycles 2–6

^3^FCR—Fludarabine was administered orally at a dose of 24 mg/m^2^ and cyclophosphamide was administered orally at a dose of 150 mg/m^2^ per day for the first 5 days of each cycle. Rituximab was administered intravenously at 375 mg/m^2^ on day 1 of cycle 1 and 500 mg/m^2^ on day 1 in cycles 2–6

^4^Obinutuzumab—100 mg on day 1, 900 mg on day 2, 1000 mg on day 8, and 1000 mg on day 15 of cycle 1 and on day 1 of subsequent 28-day cycles, for a total of six cycles

^5^Chlorambucil – 0.5 mg/kg orally on days 1 and 15 of each 28-day cycle for six cycles

^6^Bendamustine—six cycles of bendamustine (administered at a dose of 90 mg per square meter of body-surface area on days 1 and 2 of each cycle)

^7^Acalabrutinib—100 mg twice a day

^8^Venetoclax—50–400 mg once a day

^9^BCR—six cycles of treatment, with patients older than 65 years receiving intravenous bendamustine (90 mg/m^2^, days 1–2), whereas patients aged 65 years or younger received intravenous fludarabine (25 mg/m^2^, days 1–3) and intravenous cyclophosphamide (250 mg/m^2^, days 1–3). Intravenous rituximab (375 mg/m^2^, day 1 of cycle 1; 500 mg/m^2^, day 1 of cycles 2–6) was added to chemotherapy

^10^BR—bendamustine 90 mg/m^2^ will be administered as an IV infusion on Days 1 and 2 of a 28-day cycle and rituximab was administered intravenously at 375 mg/m 2 on day 1 of cycle 1 and 500 mg/m 2 on day 1 in cycles 2–6

^1^^1^Oftatumumab- 300 mg at week 1, followed by a dose of 2000 mg weekly for 7 weeks and then every 4 weeks for 16 weeks

^12^Zanubrutinib 17p deletion—160 mg twice a day

^13^Zanubrutinib—160 mg twice a day

^14^Investigator's choice (IR)—idelalisib 150 mg orally twice daily + rituximab 375 mg/m^2^ IV on day 1 of cycle 1, then 500 mg/m^2^ every 2 weeks for 4 doses, and every 4 weeks for 3 doses (total 8 infusions)

^15^Investigator's choice (BR)—bendamustine was administered at 70 mg/m 2 IV on days 1 and 2 of each 28-day cycle in combination with rituximab (375 mg/m 2 IV on day 1 of the first cycle and 500 mg/m 2 IV thereafter on day 1 of cycles 2 through 6)

^16^Ublituxmab—was given intravenously in 28-day cycles, with increasing doses during cycle 1 (≤ 150 mg on day 1, 750 mg on day 2, and 900 mg on days 8 and 15) and continuing at 900 mg on day 1 of cycles 2–6. After cycle 6, ublituximab was given at 900 mg every three cycles

^17^Placebo—ibrutinib or placebo were initiated in cycle 1 with bendamustine plus rituximab and were continued until disease progression or unacceptable toxicity

Two trials, ELEVATE-TN and Alliance A041202, included three arms and were analyzed across multiple comparisons. ELEVATE-TN compared acalabrutinib monotherapy, acalabrutinib + obinutuzumab, and chlorambucil + obinutuzumab. Alliance A041202 compared ibrutinib monotherapy, ibrutinib + rituximab, and BR. Both were analyzed in two comparisons: BTKi monotherapy vs. another comparator, and BTKi vs. BTKi + anti-CD20 (Table 1) [9, 24].

Only one trial (HELIOS) evaluated BTKi + chemotherapy versus chemotherapy alone (ibrutinib + BR vs. BR) but did not provide sufficient data for meta-analysis [27].

Table 1 summarizes the characteristics of the included studies. Fifty percent (9/18) of the trials included patients with relapsed/refractory disease.

Risk-of-bias assessment details are presented in Table 2. The sequence generation was deemed low risk in 94.45% (17/18) of trials. Allocation concealment was rated as low risk in 83.33% (15/18), and 88.89% (16/18) of studies were unblinded. All trials were considered low risk for incomplete outcome data.

**Table 2:** Risk of bias assessment

Study	Sequence generation	Allocation concealment	Blinding	Incomplete outcome data	Selective Outcome Reporting
Mohty (AMPLIFY), 2025 [26]	Low risk	Low risk	None	Low risk	Low risk
Brown (ALPINE), 2023 [7]	Low risk	Low risk	None	Low risk	Low risk
Byrd (ELEVATERR), 2021 [6]	Low risk	Low risk	None	Low risk	Low risk
Byrd (RESONATE), 2014 [5]	Low risk	Moderate risk	None	Low risk	Low risk
Burger, 2019 [21]	Low risk	Moderate risk	None	Low risk	Low risk
Burger JA (RESONATE 2), 2015 [4]	Moderate risk	Moderate risk	Triple blind	Low risk	Low risk
Chanan- Khan (HELIOS), 2016 [25]	Low risk	Low risk	Triple blind	Low risk	Low risk
Fürstenau (GAIA), 2024 [17]	Low risk	Low risk	None	Low risk	Low risk
Sharman JP (GENUINE), 2021 [22]	Low risk	Low risk	None	Low risk	Low risk
Ghia (ASCEND), 2020 [15]	Low risk	Low risk	None	Low risk	Low risk
Hillmen (FLAIR), 2023 [16]	Low risk	Low risk	None	Low risk	Low risk
Niemann (GLOW), 2023 [18]	Low risk	Low risk	None	Low risk	Low risk
Moreno (ILLUMINATE), 2019 [23]	Low risk	Low risk	None	Low risk	Low risk
Munir (FLAIR), 2023 [9]	Low risk	Low risk	None	Low risk	Low risk
Shanafelt (E1912), 2018 [24]	Low risk	Low risk	None	Low risk	Low risk
Sharman (ELEVATE-TN), 2021 [14]	Low risk	Low risk	None	Low risk	Low risk
Tam CS (SEQUOIA), 2022 [13]	Low risk	Low risk	None	None	Low risk
Woyach (ALLIANCE), 2018 [20]	Low risk	Low risk	None	None	Low risk

### Primary outcomes: any infection and grade 3–4 infection

**Fig. 3:**
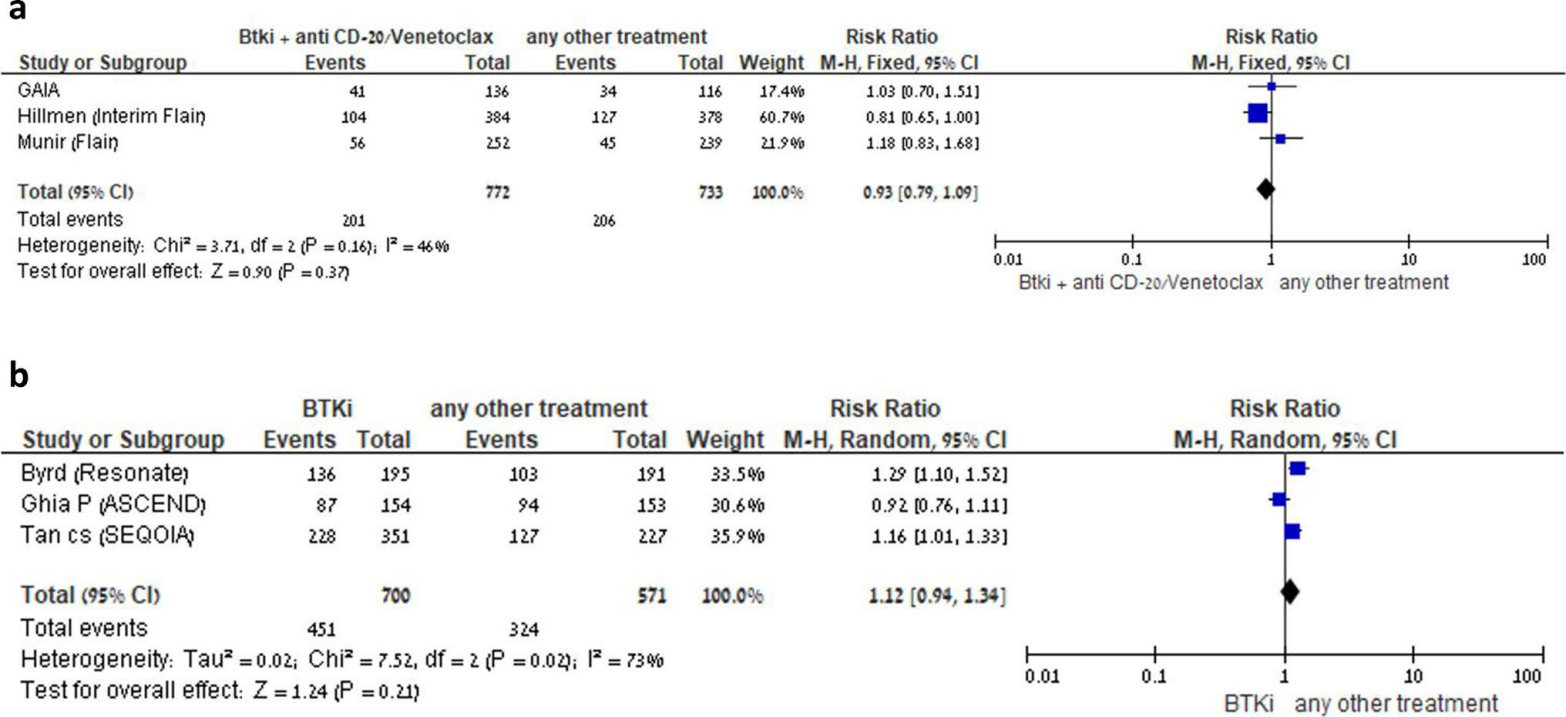
**A** BTKi with anti-CD20/venetoclax *vs* other treatment: Risk of any infection. Black squares represent the point estimate, their sizes represent their weight in the pooled analysis, and the horizontal bars represent the 95% CI. The black diamond at the bottom represents the pooled point estimate. **B** BTKi *vs* other treatment: Risk of any infection. Black squares represent the point estimate, their sizes represent their weight in the pooled analysis, and the horizontal bars represent the 95% CI. The black diamond at the bottom represents the pooled point estimate

Among the trials that compared BTKis as monotherapy *vs* another comparator, three reported the number of events of any infection [5, 13, 15]. BTKis were not associated with an increased risk of infections [RR 1.12; 95% CI 0.94–1.34; I^2^ = 73%; random-effects model, 3 trials] (Fig. 3). There were not enough data on any infections in the other five comparison groups, BTKis + anti-CD20 *vs* another comparator, BTKis + venetoclax *vs* another comparator, BTKis + anti-CD20 + venetoclax *vs* another comparator, Ibrutinib *vs* other BTKis and BTKi *vs* BTKi with anti-CD20.

**Fig. 4:**
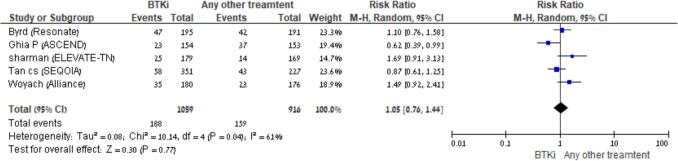
BTKi *vs* other treatment: Risk of grade 3–4 infection. Black squares represent the point estimate, their sizes represent their weight in the pooled analysis, and the horizontal bars represent the 95% CI. The black diamond at the bottom represents the pooled point estimate

Five trials that compared BTKi monotherapy *vs* another comparator reported the number of grade 3–4 infections [5, 13–15, 20]. BTKis were not associated with an increased rate of grade 3–4 infections [RR, 1.05 (95% CI; 0.76–1.44*, I*^2^ = 61%), random effects model, 5 trials] (Fig. 4). Three trials that compared BTKi *vs* BTKi with anti-CD20 reported the number of grade 3–4 infections [14, 20, 21]. BTKi with anti-CD20 were not associated with an increased rate of grade 3–4 infections compared to BTKi [RR, 1.02 (95% CI; 0.66–1.57*, I*^2^ = 58%), random effects model, 3 trials]. There were not enough data on grade 3–4 infections in the other four comparison groups, BTKis + anti-CD20 *vs* another comparator, BTKis + venetoclax *vs* another comparator, BTKis + anti-CD20 + venetoclax *vs* another comparator, Ibrutinib *vs* other BTKis.

In the HELIOS trial, there was no difference in any infections between ibrutinib + BR and BR arms (70% infections reported in both arms). Similarly, there was no significant difference in grade 3–4 infections (29% vs. 25%, respectively) [25].

#### Secondary outcomes

Data on sepsis, pneumonia, fatal infections, and febrile neutropenia were variably reported across comparison groups.

**Fig. 5:**
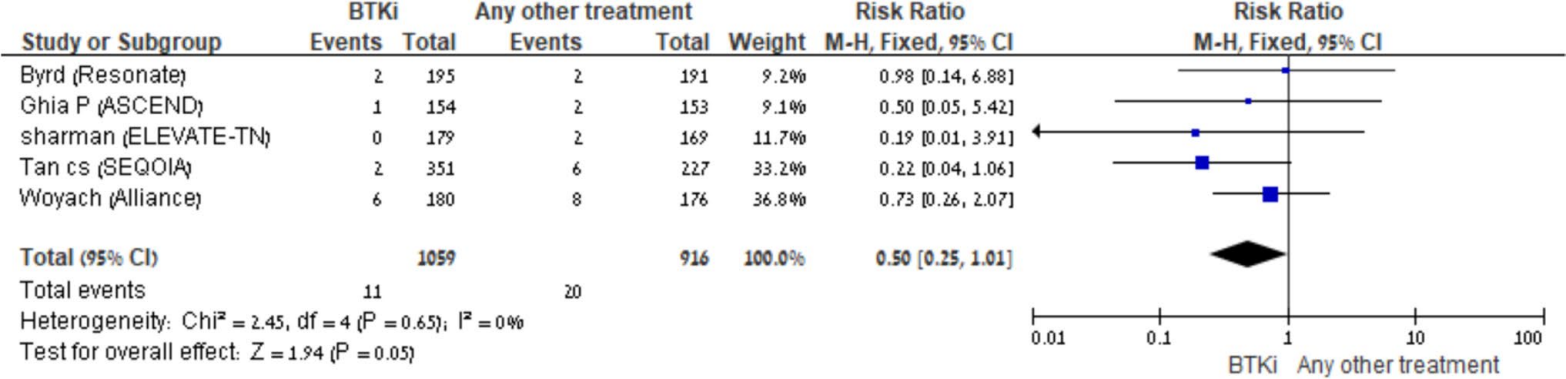
BTKi *vs* other treatment: Risk of sepsis. Black squares represent the point estimate, their sizes represent their weight in the pooled analysis, and the horizontal bars represent the 95% CI. The black diamond at the bottom represents the pooled point estimate

Sepsis was reported in three comparison groups; No significant difference was observed in the BTKi + anti-CD20 vs another comparator: RR 0.48 (95% CI: 0.12–1.84, I^2^ = 69%, 4 trials), or in the BTKi monotherapy vs another comparator: RR 0.50 (95% CI: 0.25–1.01, I^2^ = 0%, 5 trials), although the latter showed a trend toward reduced risk (Fig. 5A–B). In contrast, an increased risk was observed in the BTKi + anti-CD20 vs BTKi group: RR = 2.24; 95% CI: 0.99–5.08; I^2^ = 0%; fixed-effect model; 4 trials, suggesting a potential safety signal with combination therapy.

**Fig. 6:**
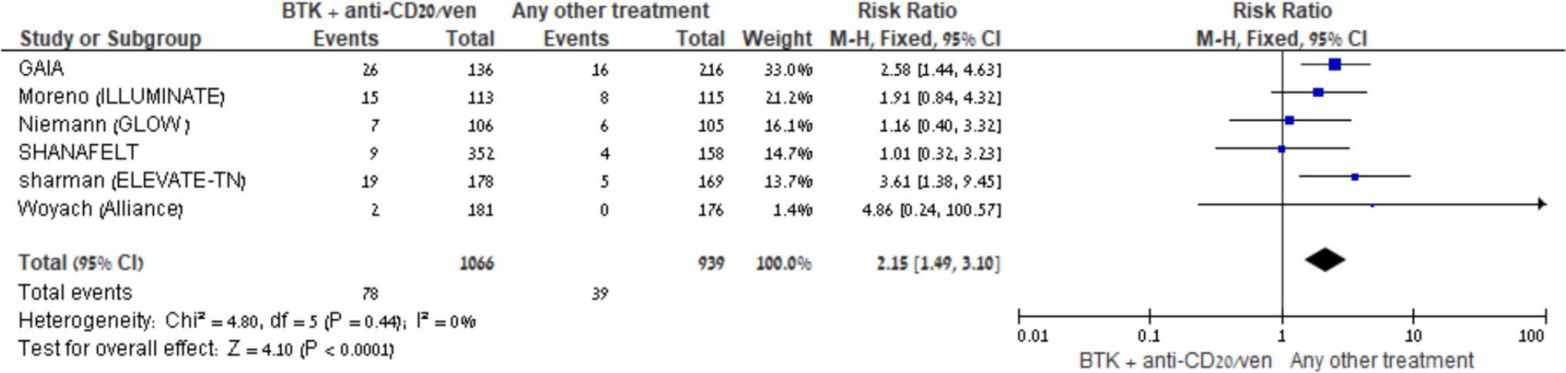
BTKi with anti-CD20/venetoclax *vs* other treatment: Risk of pneumonia. Black squares represent the point estimate, their sizes represent their weight in the pooled analysis, and the horizontal bars represent the 95% CI. The black diamond at the bottom represents the pooled point estimate

Pneumonia risk was increased in the BTKi + anti-CD20 vs another comparator group, [RR 2.18; 95% CI 1.29–3.70; I^2^ = 3%; fixed-effect model, 4 trials] as presented in Fig. 6, while no increased risk was observed in BTKi monotherapy or BTKi + anti-CD20 vs BTKi groups. Results for BTKi + venetoclax were not pooled because there were only 2 trials.

**Fig. 7:**
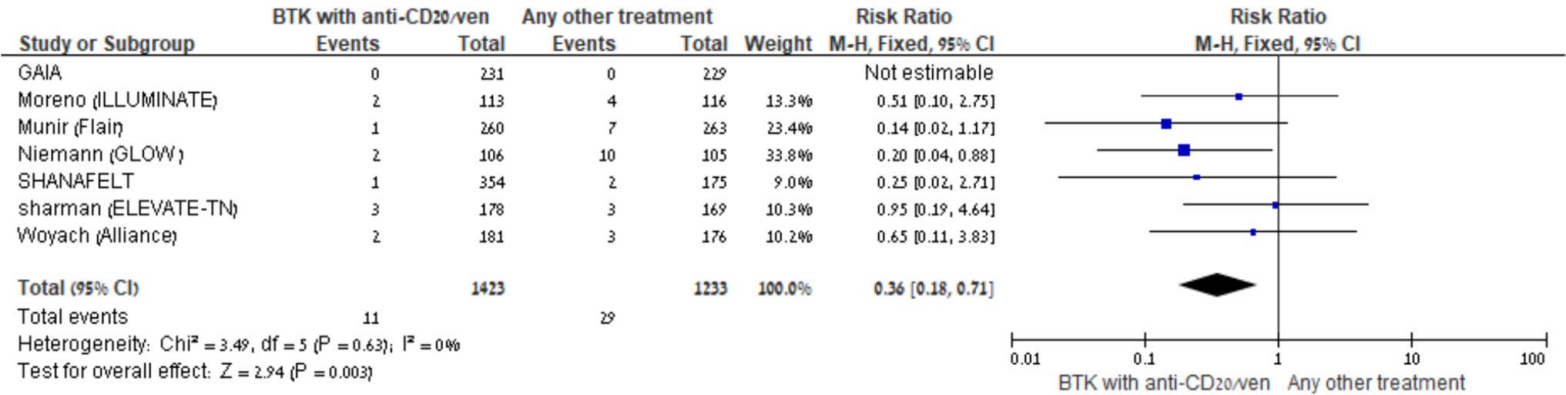
BTKi with anti-CD20/venetoclax *vs* other treatment: Risk of fatal infections. Black squares represent the point estimate, their sizes represent their weight in the pooled analysis, and the horizontal bars represent the 95% CI. The black diamond at the bottom represents the pooled point estimate

Fatal infections were reported in two comparison groups. No increased risk was observed in either BTKi + anti-CD20 vs another comparator (4 trials, Fig. 7) or BTKi monotherapy vs another comparator (4 trials).

**Fig. 8:**
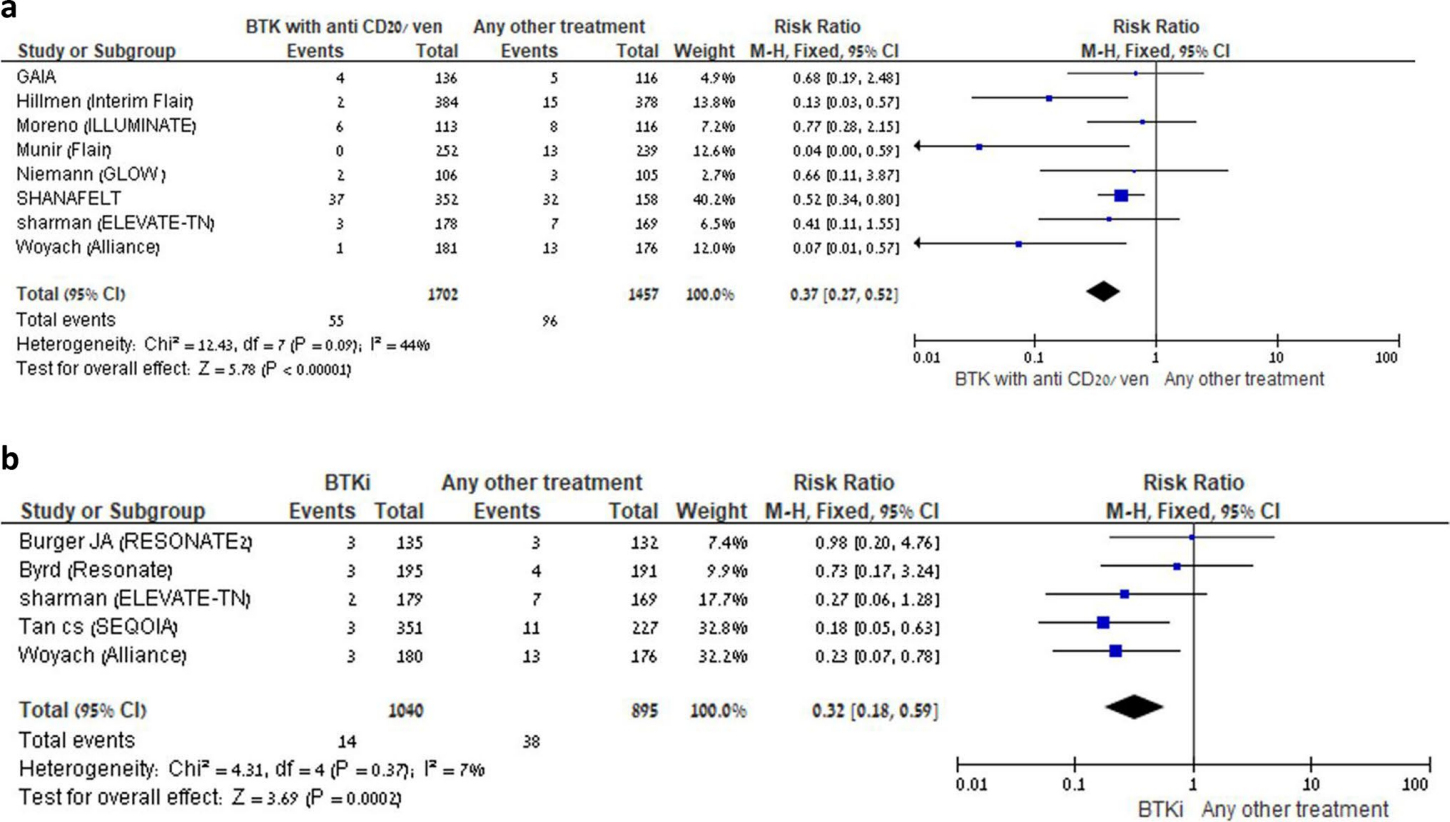
**A** BTKi with anti-CD20/venetoclax vs other treatment: Risk of neutropenic fever. Black squares represent the point estimate, their sizes represent their weight in the pooled analysis, and the horizontal bars represent the 95% CI. The black diamond at the bottom represents the pooled point estimate. **B** BTKi *vs* other treatment: Risk of neutropenic fever. Black squares represent the point estimate, their sizes represent their weight in the pooled analysis, and the horizontal bars represent the 95% CI. The black diamond at the bottom represents the pooled point estimate

Febrile neutropenia was consistently reduced in all three groups where data were available: BTKi + anti-CD20 vs another comparator (RR 0.41, 95% CI:0.29–0.59, I^2^ = 38%; 5 trials), BTKi + venetoclax vs another comparator (RR 0.18, 95% CI: 0.08–0.40, I^2^ = 41%; 3 trials), and BTKi monotherapy vs another comparator (RR 0.32, 95% CI:0.18–0.59, I^2^ = 7%; 5 trials) (Fig. 8A–B).

There were insufficient data to assess these outcomes in the remaining comparison groups.

## Discussion

In this systematic review and meta-analysis, which included 18 randomized controlled trials (RCTs) with 8,324 patients, we assessed the risk of infections associated with BTKi-containing regimens in the treatment of CLL/SLL. Our findings indicate that BTKi, whether as monotherapy or combined with anti-CD20, are not associated with an increased risk of overall infections or grade 3–4 infections compared to chemotherapy or chemoimmunotherapy. Additionally, the risk of pneumonia was increased when BTKi were combined with anti-CD20, while febrile neutropenia occurred less frequently with BTKi-containing regimens compared to chemotherapy.

The main finding is not surprising, as the comparator in most trials included chemotherapy, most commonly combined with an anti-CD20. The lack of increased risk of infections and grade 3–4 infections with BTKi regimens is consistent with a previous systematic review by Mato et al. [9], which analyzed four RCTs comparing ibrutinib to other regimens. That review included 756 patients treated with ibrutinib and 749 treated with comparators such as ofatumumab, chlorambucil, and bendamustine-based regimens. It reported infection rates of 70% for ibrutinib-treated patients versus 63% for comparators, with grade 3–4 infections occurring in 23% and 22%, respectively. Our broader analysis, which includes additional BTKi agents and combinations, aligns with these findings of no increased risk, and further supports the safety of BTKi in this context.

An important observation from our analysis is the increased risk of pneumonia associated with BTKi combined with anti-CD20 or as monotherapy, compared to chemotherapy or chemoimmunotherapy. Interestingly, this increased risk was not observed with BTKi monotherapy, suggesting that the addition of anti-CD20 may contribute to respiratory complications. Previous studies have highlighted the high incidence of pneumonia in patients treated with ibrutinib, with a systematic review showing that approximately 20% of patients developed pneumonia, regardless of whether ibrutinib was used as a single agent or in combination therapy [11]. Notably, ibrutinib-induced pneumonitis has also been reported [28], raising the possibility that some cases of pneumonia attributed to infection may be due to drug-induced pulmonary toxicity.

We found that the risk of neutropenic fever was lower in both BTKi groups compared to other chemotherapy regimens. This finding is not surprising since chemotherapy usually carries a high risk of febrile neutropenia [27]. Grade ≥ 3 neutropenia is common in the first months of treatment with ibrutinib, occurring in 10–17% of patients [29, 30]. It's crucial to emphasize that the risk of febrile neutropenia was not higher despite the high rates of neutropenia previously described.

The strength of our study stems from the fact that it is the largest systematic review and meta-analysis that includes all available RCTs that evaluated any BTKi combination, in all reported comparisons. In addition, all BTKi were included and not just ibrutinib. In addition, our review included trials of both first line and relapsed patients.

Despite its strengths, our study has limitations that merit discussion. The included trials were heterogeneous in terms of comparator regimens, patient populations, and study designs. This heterogeneity limited our ability to perform subgroup analyses by disease stage (first-line vs. relapsed/refractory) or BTKi type. Additionally, data on the pathogens responsible for pneumonia (bacterial, viral, or fungal) were scarce, making it difficult to draw definitive conclusions about the microbiological aspects of infections in this patient population. Moreover, most comparators in the included trials were chemotherapy-based regimens, which may not reflect the current treatment landscape dominated by non-chemotherapy options such as venetoclax-based therapies.

The inclusion of non-chemotherapy regimens, such as venetoclax combined with obinutuzumab, in current first-line treatment guidelines highlights the need for updated comparisons. However, our systematic search did not identify any head-to-head randomized controlled trials comparing BTKi-based regimens with venetoclax-obinutuzumab that met the inclusion criteria for this meta-analysis. Retrospective studies, such as the one comparing BTKi with venetoclax-obinutuzumab, have not reported infections as an outcome, leaving significant gaps in the evidence base [31]. Similarly, while the Murano trial compared venetoclax-rituximab to chemotherapy in relapsed settings, data on infection outcomes with venetoclax-rituximab versus BTKi regimens are lacking [32]. In a Bayesian network meta-analysis, venetoclax-rituximab was associated with lower rates of grade ≥ 3 infections compared to acalabrutinib, ibrutinib, and zanubrutinib, potentially due to the shorter treatment duration of venetoclax-based regimens [33]. This finding warrants further exploration in future RCTs.

Emerging treatment strategies, such as fixed-duration ibrutinib-venetoclax combinations, represent an important shift in the therapeutic paradigm for CLL. While ongoing trials such as CLL17 aim to compare continuous versus fixed-duration BTKi regimens, data on infection outcomes remain limited [34]. Similarly, trials assessing combinations of BTKi with venetoclax and anti-CD20 antibodies, such as the MAJIC and CLL16 trials, will be instrumental in determining whether these regimens improve efficacy without compromising safety [35].

In conclusion, our meta-analysis demonstrates that BTKi regimens have a comparable overall infection risk to chemotherapy in patients with CLL/SLL, but the addition of anti-CD20 or venetoclax to BTKi is associated with an increased risk of pneumonia. As treatment paradigms evolve, infection risk should remain a key consideration in therapeutic decision-making. Future trials should prioritize head-to-head comparisons of BTKi-based regimens with other non-chemotherapy options, focusing on infection outcomes and optimal treatment duration to guide evidence-based clinical practice.

## Supplementary Information

Below is the link to the electronic supplementary material.

Supplementary file1 (DOCX 175 KB)

Supplementary file2 (DOCX 270 KB)

## Data Availability

No datasets were generated or analysed during the current study.
