# Supplementary material for: Infectious complications in CLL/SLL patients receiving Bruton's Tyrosine Kinase inhibitors – systematic review and meta-analysis of randomized controlled trials
Source: Ann Hematol. 2025 Jul 25;104(8):3903–15. doi: 10.1007/s00277-025-06502-y (PMC12432036; doi:10.1007/s00277-025-06502-y)
Supplement: Supplementary file 1 — Supplementary file1 (DOCX 175 KB) [file 277_2025_6502_MOESM1_ESM.docx]

#### **Legends to Figures**

**Figure 1** - Trial flow according to Preferred Reporting Items for Systematic Reviews and Meta-Analyses (PRISMA), showing flow of trials included in the meta-analysis.

**Figure 2** – Classification of Included Trials According to Treatment Comparisons

**Figure 3** – BTKi *vs* other treatment: Risk of any infection. Black squares represent the point estimate, their sizes represent their weight in the pooled analysis, and the horizontal bars represent the 95% CI. The black diamond at the bottom represents the pooled point estimate.

**Figure 4** – BTKi *vs* other treatment: Risk of grade 3-4 infection. Black squares represent the point estimate, their sizes represent their weight in the pooled analysis, and the horizontal bars represent the 95% CI. The black diamond at the bottom represents the pooled point estimate.

**Figure 5A** – BTKi + anti-CD20 *vs* other treatment: Risk of sepsis. Black squares represent the point estimate, their sizes represent their weight in the pooled analysis, and the horizontal bars represent the 95% CI. The black diamond at the bottom represents the pooled point estimate.

**Figure 5B** – BTKi *vs* other treatment: Risk of sepsis. Black squares represent the point estimate, their sizes represent their weight in the pooled analysis, and the horizontal bars represent the 95% CI. The black diamond at the bottom represents the pooled point estimate.

**Figure 6** – BTKi with anti-CD20 *vs* other treatment: Risk of pneumonia. Black squares represent the point estimate, their sizes represent their weight in the pooled analysis, and the horizontal bars represent the 95% CI. The black diamond at the bottom represents the pooled point estimate.

**Figure 7** – BTKi with anti-CD20 *vs* other treatment: Risk of fatal infections. Black squares represent the point estimate, their sizes represent their weight in the pooled analysis, and the horizontal bars represent the 95% CI. The black diamond at the bottom represents the pooled point estimate

**Figure 8A** – BTKi with anti-CD20 vs other treatment: Risk of neutropenic fever. Black squares represent the point estimate, their sizes represent their weight in the pooled analysis, and the horizontal bars represent the 95% CI. The black diamond at the bottom represents the pooled point estimate.

**Figure 8B** – BTKi *vs* other treatment: Risk of neutropenic fever. Black squares represent the point estimate, their sizes represent their weight in the pooled analysis, and the horizontal bars represent the 95% CI. The black diamond at the bottom represents the pooled point estimate.

***Figure 1:*** *PRISMA Flow Diagram*

Potentially relevant trials identified and screened for retrieval in

(n=2,641)

Additional records identified through relevant conference abstract book searching
(n=18)

Records screened after duplicates removed

(n=2,659)

Records excluded by title, irrelevant for study question

(n =2,629)

Full-text articles assessed for eligibility

(n=30)

Full-text articles and abstracts excluded, (n = 12):

Not infectious outcomes reported, n= 7

Missing results, n=5

Studies included in qualitative synthesis

**(n=18)**

## Identification

## Screening

## Eligibility

## Included

**Figure 2:** Classification of Included Trials According to Treatment Comparisons

***Figure 3:*** *Any Infection – BTKi vs Others*


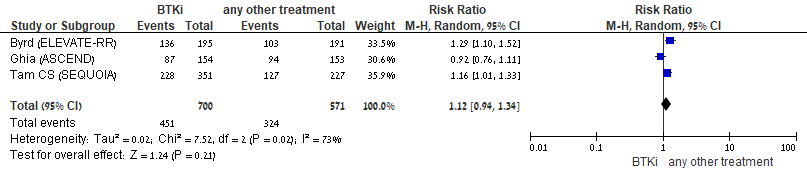


***Figure 4:*** *Grade 3–4 Infections – BTKi vs Others*


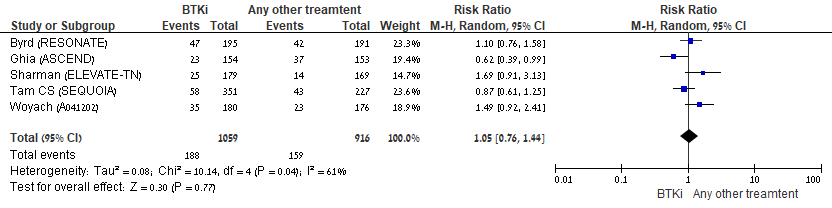


***Figure 5A:*** *Sepsis – BTKi vs Others*


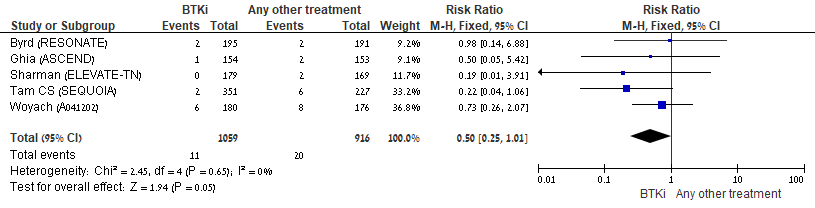


***Figure 5B:*** *Sepsis – BTKi+anti-CD20 vs Others*


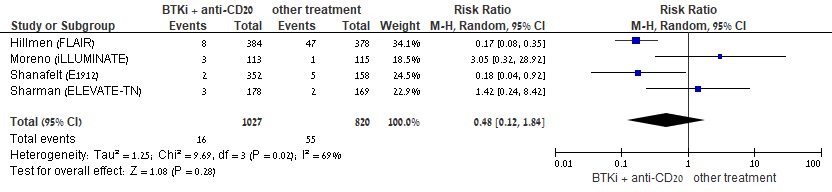


***Figure 6:*** *Pneumonia Risk – BTKi + anti-CD20 vs Others*

**
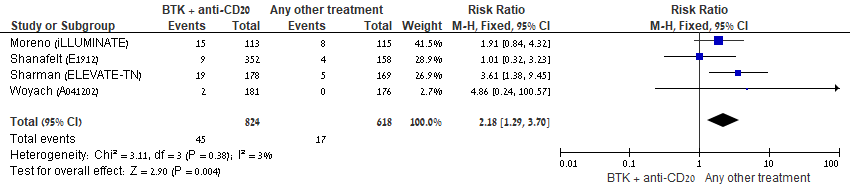
**

***Figure 7:*** *Fatal Infections – BTKi + anti-CD20 vs Others*

**
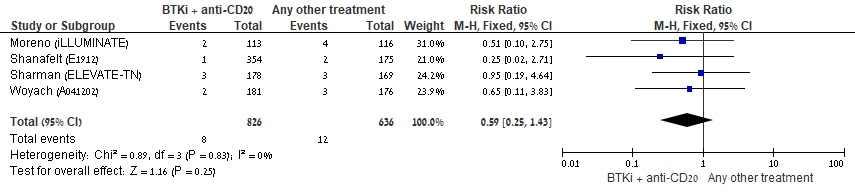
**

***Figure 8A:*** *Neutropenic Fever – BTKi + anti-CD20 vs Others*

**
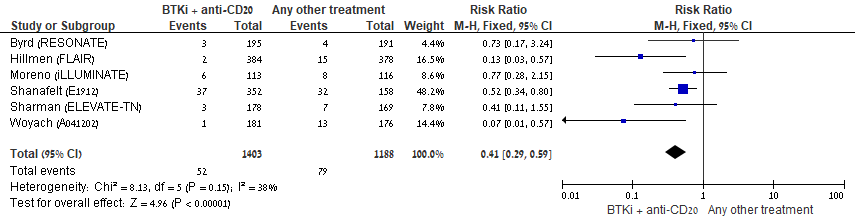
**

***Figure 8B:*** *Neutropenic Fever – BTKi vs Others*

**
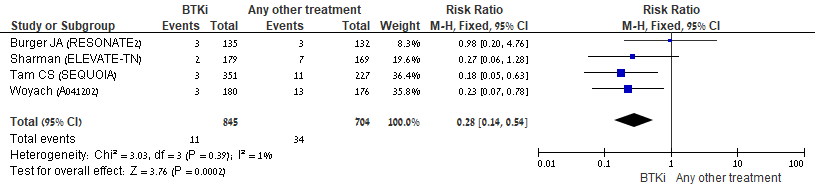
**
